# Level of evidence for promising subgroup findings in a negative trial

**DOI:** 10.1186/1745-6215-14-S1-O105

**Published:** 2013-11-29

**Authors:** Julien Tanniou, Ingeborg van der Tweel, Kit Roes

**Affiliations:** 1 UMC Utrecht, Julius Center, Utrecht, The Netherlands

## 

In drug development and drug licensing, it sometimes occurs that a new drug did not demonstrate effectiveness for the full study population, but there appears to be benefit in a relevant subgroup. This raises the question how strong the evidence from such a subgroup is in this situation, and which confirmatory testing strategies are the most appropriate.

Using simulation studies, we compared the evolution of the type I error rate and the power of several types of confirmatory strategies, including replications of subgroup findings in parallel or new studies. The primary outcome was assumed to be continuous and normally distributed.

In case of a single trial, the inflation of the overall type I error is substantial, especially in relatively small subgroups with the added risk of starting a replication trial that should not be done. When the p-value for the subgroup of interest is very small, i.e. below 0.001, the strength of evidence is improved but such a strong result is not likely to occur in case of an overall non-significant result. The level of evidence improves as expected when subgroup findings are replicated in (the design of) a new trial. In this situation, the overall type I error is very close to the theoretical value, and almost guarantees its control.

